# Smartphone accelerometer data as a proxy for clinical data in modeling of bipolar disorder symptom trajectory

**DOI:** 10.1038/s41746-022-00741-3

**Published:** 2022-12-14

**Authors:** Casey C. Bennett, Mindy K. Ross, EuGene Baek, Dohyeon Kim, Alex D. Leow

**Affiliations:** 1 Department of Intelligence Computing, Hanyang University, Seoul, Korea; 2 Department of Computing, DePaul University, Chicago, IL USA; 3 Department of Psychiatry, University of Illinois–Chicago, Chicago, IL USA; 4 Dept. of Biomedical Engineering, University of Illinois–Chicago, Chicago, IL USA

**Keywords:** Bipolar disorder, Predictive markers, Machine learning, Computational science

## Abstract

Being able to track and predict fluctuations in symptoms of mental health disorders such as bipolar disorder outside the clinic walls is critical for expanding access to care for the global population. To that end, we analyze a dataset of 291 individuals from a smartphone app targeted at bipolar disorder, which contains rich details about their smartphone interactions (including typing dynamics and accelerometer motion) collected everyday over several months, along with more traditional clinical features. The aim is to evaluate whether smartphone accelerometer data could serve as a proxy for traditional clinical data, either by itself or in combination with typing dynamics. Results show that accelerometer data improves the predictive performance of machine learning models by nearly 5% over those previously reported in the literature based only on clinical data and typing dynamics. This suggests it is possible to elicit essentially the same “information” about bipolar symptomology using different data sources, in a variety of settings.

## Introduction

Recent years have seen an explosion of researchers using smartphones to understand patterns of user behavior and their relationship to chronic health conditions^1–3^, including in particular typing dynamics for conditions such as bipolar disorder^4,5^. Typing dynamics refers to the speed and rhythm with which users type on their phone (e.g., when sending emails or text messages or posting to social media), which can be captured in various metrics that describe things such as the transition time between keypresses, repetitive presses of the same key, press duration, use of backspace and autocorrect, and so on. Patterns in those metrics are thought to potentially relate to a person’s underlying physiological and cognitive state, and, thus, by extension, to their current health status. Indeed, previous research has shown how such typing dynamics can predict fluctuations of symptoms in conditions such as bipolar disorder with a high degree of accuracy^6–8^.

However, an open question remains as to the integration of other sensor data from smartphones with typing dynamics, e.g. accelerometer data. Would such data enhance the predictive performance of the aforementioned models, or could it even serve as a substitute for typing dynamics or traditional clinical data? Accelerometer data is of particular interest for mental health conditions such as bipolar disorder, where psychomotor disturbances leading to periods of increased movement (agitation) or decreased movement (impairment) can be notable symptoms^9^. For instance, during psychomotor agitation, those disturbances can manifest as pacing, fidgeting, shaking, etc., which could theoretically correspond to changes in phone movement during use. Similar patterns may also be present in other disorders with psychomotor disturbances, such as dementia^10^.

Some research has recently begun to explore similar questions related to multimodal data for use in predicted trajectories of chronic health conditions outside the clinic walls^11,12^, but it is often thus far limited in scope, both in terms of the types of sensors explored and the setting (e.g., controlled study vs. real-world). There are many possible sensor data sources that could be incorporated, in many different ways, either potentially as new separate features in the dataset or through sensor fusion methods to enhance existing typing dynamics features via the creation of fused features^13^. In short, **understanding how all those possible data sources might be integrated with typing dynamics requires isolating the effects of each source** and carefully studying the impact on overall model performance in real-world settings where user behavior is unconstrained and the sensor data often very messy. Our purpose here is to do just that, focusing on the integration of accelerometer data with typing dynamics for predicting fluctuations in bipolar symptoms outside the clinic using a crowdsourced open-science dataset.

Furthermore, those above questions have even broader applicability if one considers the inclusion of sensor data from devices beyond the smartphone itself, such as wearables, in-home robotics, and other smart home technology, which could communicate with the smartphone as part of internet-of-things (IOT) systems. The same methods and approaches for studying multimodal smartphone data could then be extended to those other types of data sources, in order to understand the impact of multi-device sensing on the modeling of chronic health conditions relative to existing traditional clinical data. As many societies seek to expand healthcare into community-based settings to address issues like a growing elderly population, increased demand for mental health services, and limited health resources, research along these lines takes on fundamental importance^14,15^. The true power of many technologies likely lies at the intersection of using them in concert, integrating various types of sensor data, and providing back actionable information to patients and clinicians. The question is how we do so.

One of the defining features of bipolar disorder, averse to other affective disorders, is reoccurring manic/hypomanic and depressive episodes^16^. Individuals with this disorder experience repeated changes in mood, ranging between clinical definitions of mania and depression, as well as subclinical fluctuations that occur on a more rapid basis. Such mood instability has significant implications for both treatment and patient outcomes in bipolar disorder^17,18^, as well as understanding the underlying etiology of the disease^19^.

Given the above, mood instability has been one of the research targets for developing digital biomarkers for bipolar disorder in recent years^6^. For instance, previous research has found significantly higher mood instability in the 60 days leading up to a clinical event of depression or mania^20^. Such findings have led to work that attempted to predict bipolar status based on device sensor data, such as smartphone geolocation data^21^, as well as daily activity patterns from wearable smartwatches^22^. Some research has also investigated technology interaction with the device itself (rather than trying to sense external activity) as a proxy for mental health status, using a range of technology, including smartphones, wearables, and other mobile devices^23^. Such approaches fall into the category of *ambulatory assessment*^24^. Other researchers have developed smartphone apps for patient daily self-reports^25,26^, while still others have explored real-time self-report assessment repeatedly throughout the day using techniques known as *ecological momentary assessment* *(*EMA)^6^. In short, there are a plethora of different approaches being explored. Passive smartphone data (e.g., typing dynamics and accelerometer data) is of particular use for this purpose, as it can be collected in a completely unobtrusive manner based on a device people normally use every day anyway, in contrast to wearables (e.g., Apple Watch) that may require users to consistently use an additional device that they normally don’t or methods based on GPS or voice recognition that may present more serious privacy concerns^27^.

**Our primary research aim here is to evaluate whether smartphone accelerometer data could serve as a proxy for traditional clinical data** (e.g., similar to what one might find in an electronic health record, or EHR) in the management and treatment of the bipolar disorder, both by itself or in combination with typing dynamics from keyboard interactions with the phone. Furthermore, we are interested in whether accelerometer data would enhance the predictability of mood instability, beyond what has been previously observed using clinical data and typing dynamics. To that end, we focus here on predicting changes in depressive symptoms in bipolar disorder based on data in the weeks *prior* to the change, in order to allow for a direct comparison to previous research results^6–8^. Such depressive symptoms have been found to dominate illness presentation and influence functional outcomes more than manic symptoms^18,28^, so digital biomarkers related to them are of particular relevance. Furthermore, we utilize a naturalistic dataset of users that are typically seen in real-world clinical settings, rather than a controlled study, meaning that it includes the kinds of “messy” data seen in those settings.

As alluded to in the Background section above, our broader aim here is to understand how different approaches to the in-the-wild real-time assessment of mental health disorders can be best realized via different data sources and multiple technologies. Understanding the subtle differences in different types of smartphone data for that purpose is a necessary first step towards integrating smartphones with similar real-time monitoring data of patient daily activities from technologies such as smart home IOT devices and in-home robotics^29–31^.

## Results

### Main results

The data used here consisted of an open-science dataset of 291 individuals who downloaded the BiAffect smartphone app from the Apple Appstore between Spring 2018 and Spring 2021. On average, we had roughly 3 months of data for each individual. After downloading, the BiAffect app substitutes a cosmetically similar keyboard in place of the standard iOS keyboard, which allows it to record keystroke dynamics metadata regardless of how the phone is used (e.g., texting, writing an email, posting on social media, etc.)^4^. Simultaneously, accelerometer data from the phone was also being recorded alongside the typing dynamics metadata whenever the keyboard was in use. This allows us to collect pervasive data on passive technology interactions and their relationship to phone movement in 3-dimensional space over an extended period of time. Restricting accelerometer data to only periods of keyboard use allowed us to avoid the known problem in the field of human activity recognition of “noisy” data due to users carrying their phones in different orientations (e.g., on its side in their jacket pocket or purse rather than upright in their pants pocket, laying in the cupholder while driving)^32–34^. In our dataset, we found that users' phones were in an approximately upright position while typing, typically about 80% of the time.

**Fig. 1:**
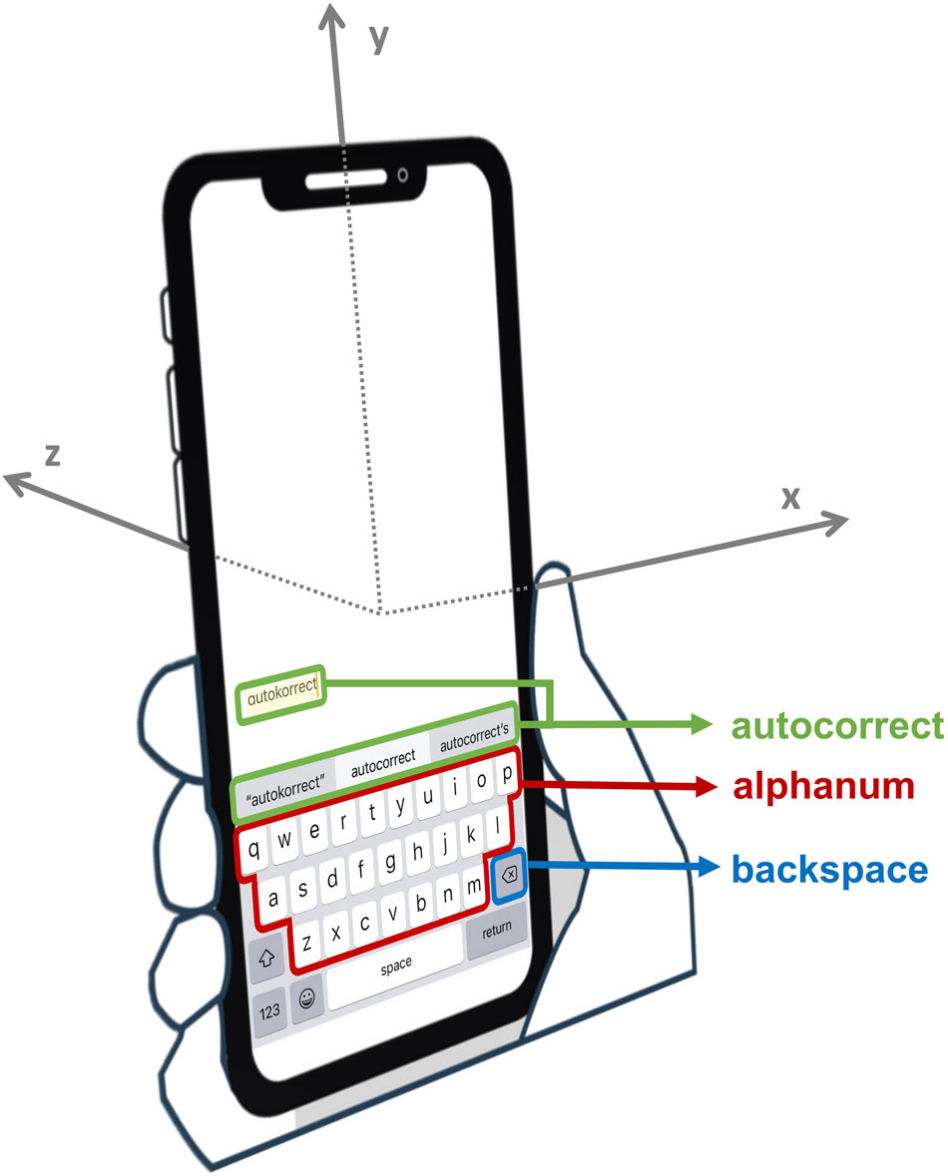
Visual diagram of accelerometer coordinate system. Note that each coordinate is bidirectional along its axis, e.g., the z-axis can entail moving the phone *away* from oneself but also moving it *towards* oneself.

Dataset features are shown in Table 1, grouped by category (also see Fig. 1 below). We note that included traditional clinical data, such as diagnoses, standardized clinical symptom measures (e.g., MDQ), and demographic variables like age and gender. Each week, users were also pinged to complete several types of self-report assessments on a daily or weekly basis. That included a weekly PHQ outcome scale, which is a widely used measure of depression symptoms^35^. Approximately 2/3 of the individuals reported having been diagnosed bipolar, whereas others were undiagnosed (who may or may not have bipolar). Our dataset thus contains both diagnosed and potential cases of bipolar disorder in the general population. The aim was to predict clinically-relevant changes in PHQ scores for a given week *before* they occur, based on smartphone interaction data in the weeks prior.

**Table 1:** Feature list.

Category	Data	Description
Clinical	Age, gender	User’s age/gender
	phoneSize	Scale of phone size based on phone model
	ADHD, anxiety, BD_binary, depression, OCD, PTSD, schizophrenia, seasonal affective disorder, substance addiction disorder	Diagnosis of ADHD/anxiety/bipolar disorder/depression /OCD/PTSD/schizophrenia/seasonal affective disorder/substance addiction disorder
	Diag_PreferNotAnswer	Preferred not to respond to diagnosis questions
	MDQdiag	MDQ diagnosis
	NoneOfTheseDiag	None of the listed diagnoses
	PHQ_before_1w	Absolute value of the PHQ8 score from weeks prior
Typing dynamics	autocorrectRate, backspaceRate	Fraction of autocorrect events / backspaces per total keypresses for each week
	autocorrectRate_wkSD, bkspRate_wkSD, medIKD_wkSD	Standard deviation of autocorrect rates/backspace rates/median IKDs for each day over the week
	Avg_medPressDuration	Median keypress duration for each typing session averaged over the week
	Avg_nAlphanum, Avg_nAutocorrect, Avg_nBackspace	Number of alphanum/autocorrect/backspace keypresses per session averaged over each week
	Avg90PercentileAA	Ninetieth percentile of alphanum-alphanum transitions for each typing session averaged over the week
	AvgMAD_AA	Median absolute deviation of alphanum-alphanum transitions for each typing session averaged over the week
	AvgMedAA, AvgVarAA	Median/variance of alphanum-alphanum transition for each typing session averaged over the week
	AvgMedAB, AvgvarAB	Median/variance of alphanum-backspace transition for each typing session averaged over the week
	AvgMedBB, AvgVarBB	Median / variance of backspace-backspace transition for each typing session averaged over the week
	distToCenterPrevRatioAA	Median ratio of distance to center of key and distance to the previous keypress for alphanum-alphanum transitions for each week
	medianDistCenter	Median distance from touch to center of key for each week
	medianIKD	Median IKD for each week
	medianPressDur	Median keypress duration for each week
	nKeypresses	Number of keypresses per week
Accelerometer	arc_sum	3D Rotational motion per week (calculated based on X/Y/Z accelerometer readings)
	count_Xhorizontal	Number of X readings that were greater than ± 0.8 per week (i.e. number of times phone was in a horizontal position to the ground)
	medianX, medianY, medianZ	Median X/Y/Z accelerometer readings per week
	n_XYZ	Number of accelerometer readings per week, indicating the number of times motion was detected (based on “sensor events”)
	Xmotion_sum, Ymotion_sum, Zmotion_sum	Sum of differences between consecutive X/Y/Z readings per week
	Xmotion_sd, Ymotion_sd, Zmotion_sd	Standard deviation of differences between consecutive X/Y/Z readings per week

The main results of our analysis can be seen in Table 2 (with mean imputation) and Table 3 (excluding missing data). In general, we note that the differences between imputation and exclusion were small and of mixed effect. Given that fact and since imputation left us with an overall larger dataset, that method was chosen for the rest of the analysis presented here.

**Table 2:** Main results with mean imputation^a^.

Features (# of features)	Classifier	Acc	Acc std	AUC	AUC std	Sensitivity
All (50)	Random Forest	0.9450	0.03	0.9912	0.01	0.9391
	Gradient Boost	0.9377	0.06	0.9810	0.03	0.9077
	Neural Network	0.9275	0.01	0.9751	0.01	0.9844
Typing+Accel (34)	Random Forest	0.9134	0.01	0.9442	0.01	0.9119
	Gradient Boost	0.8836	0.02	0.9430	0.02	0.8972
	Neural Network	0.8907	0.02	0.9456	0.02	0.9119
Typing (22)	Random Forest	0.8998	0.02	0.9403	0.01	0.9035
	Gradient Boost	0.8778	0.02	0.9398	0.01	0.8888
	Neural Network	0.8871	0.02	0.9332	0.02	0.8969
Accel (12)	Random Forest	0.8704	0.02	0.9328	0.02	0.9119
	Gradient Boost	0.8395	0.03	0.9042	0.02	0.8919
	Neural Network	0.8623	0.01	0.9114	0.02	0.8854

^a^*Acc* accuracy, *AUC* area under curve, *std* standard deviation.

**Table 3:** Main results excluding missing data^a^.

Features (# of features)	Classifier	Acc	Acc std	AUC	AUC std	Sensitivity
All (50)	Random Forest	0.9211	0.06	0.9752	0.04	0.9228
	Gradient Boost	0.8898	0.09	0.9697	0.05	0.8824
	Neural Network	0.8555	0.03	0.9321	0.04	0.9653
Typing+Accel (34)	Random Forest	0.8952	0.04	0.9768	0.02	0.9559
	Gradient Boost	0.8713	0.05	0.9642	0.03	0.9338
	Neural Network	0.8670	0.05	0.9224	0.06	0.9563
Typing (22)	Random Forest	0.9155	0.03	0.9774	0.01	0.9375
	Gradient Boost	0.8861	0.04	0.9584	0.02	0.9485
	Neural Network	0.8691	0.04	0.9383	0.04	0.9512
Accel (12)	Random Forest	0.8694	0.03	0.9512	0.01	0.9228
	Gradient Boost	0.8272	0.02	0.9083	0.01	0.9044
	Neural Network	0.8545	0.04	0.9183	0.04	0.9168

^a^*Acc* accuracy, *AUC* area under curve, *std* standard deviation.

The tables clearly show that the performance gradually decreases as we go from All features (slice 1, see Methods section) down to Accelerometer only (slice 4), though we note the changes were slight. We also note that Random Forest models tended to show about 2–4% higher accuracy and slightly higher AUC values than Gradient Boosting models. Curiously, the standard deviations also reduced as we moved down to smaller feature slices (e.g., using only typing or accelerometer features), which we interpreted as due to a reduction in “noise” in the dataset after removing other features.

**Overall, the inclusion of accelerometer features here with clinical features and typing dynamics improves performance over models created without accelerometer features reported previously in many papers by roughly 4.5% (94.5 vs ~90%)**^6–8^. Statistically speaking across the five cross-validation folds (standard deviations of 0.03 and 0.02, respectively), those performance values are significantly different in a two-tailed, independent-samples *t*-test (*t* = 2.79, *p* value = 0.0235). That difference includes reported results using both traditional machine learning models (e.g., Random Forest, Gradient Boosting) as well as various types of deep learning neural networks. This indicates that there is value in utilizing smartphone accelerometer data during user interactions to predict bipolar symptom prognosis that goes beyond the direct interaction data itself (i.e., typing dynamics) or clinical data. Moreover, we also note that the combination of typing dynamics and accelerometer data achieved a performance of over 91.3% accuracy in making those same predictions, which could be useful for monitoring patients outside clinical settings (where clinical data may be unavailable or not recent). Finally, the results of the accelerometer data alone show that it can still be used to predict bipolar symptoms with roughly 87% accuracy. While that is a decrease from models including other types of data, it is indicative of the potential for using accelerometer data to monitor everyday chronic health conditions, which could have wide applicability to other technologies, not just smartphones but wearable devices or in-home robotic companions. We return to this idea in the Discussion section.

### Feature selection

We were also curious as to which features were being selected when using the different slices of the dataset features (as described in the Methods section). In particular, we were interested in whether the utility of some of the accelerometer and typing features were being “obscured” by the clinical features, due to the fact that the information contained within them relative to the target (bipolar symptom fluctuations) was duplicative. If, in fact, accelerometer features could serve as a proxy for clinical data (as suggested by the results in the Main Results section above), then we would indeed expect the information contained within them to be duplicative. In simpler terms, we should be able to produce multiple feature sets that are capable of similar modeling performance by restricting the input features.

To evaluate this, we attempted a variety of feature selection methods on those slices, which we summarize here for brevity. Table 4 shows the results of the feature rankings extracted by the filter method using information gain, based on the same slices shown in Tables 2 and 3 above. One can see that there are significant shifts in which features are “important” based on this approach. One clear example is nKeypresses, which was relatively unimportant when clinical features or accelerometer features are included, but became one of the top two most important features when only using typing features. We can see similar effects on other typing features, such as autocorrect and backspace rates, as well on some of the accelerometer features medianX and medianZ.

**Table 4:** Feature rankings by data slice^a^.

	All	Typing + Accel	Typing	Accel
1	PHQ_before_1w	***medianPressDur***	medianPressDur	n_XYZ
2	Age	***medianX***	nKeypresses	count_Xhorizontal
3	phoneSize	Xmotion_sd	bkspRate_wkSD	arc_sum
4	MDQdiag	***arc_sum***	Avg_nAutocorrect	medianX
5	Gender	n_XYZ	AvgVarAB	Ymotion_sum
6	medianPressDur	autocorrectRate_wkSD	autocorrectRate_wkSD	medianZ
7	BD_binary	medianIKD	medIKD_wkSD	Xmotion_sum
8	Depression	Avg_nAlphanum	medianIKD	Zmotion_sd
9	PTSD	medianDistCenter	backspaceRate	Ymotion_sd
10	Anxiety	medIKD_wkSD	medianDistCenter	Zmotion_sum
11	count_Xhorizontal	bkspRate_wkSD	Avg_nBackspace	Xmotion_sd
12	ADHD	AvgMedAA	distToCenterPrevRatioAA	medianY
13	SubstanceAddictionDisorder	***backspaceRate***	AvgMedAA	
14	n_XYZ	Avg_nAutocorrect	AvgVarBB	
15	arc_sum	***autocorrectRate***	AvgMAD_AA	
16	OCD	AvgVarAB	autocorrectRate	
17	medianDistCenter	AvgMedBB	Avg90PercentileAA	
18	Avg_nBackspace	AvgVarBB	Avg_nAlphanum	
19	backspaceRate	Avg_medPressDuration	AvgVarAA	
20	nKeypresses	Zmotion_sum	AvgMedBB	
21	AvgMAD_AA	Ymotion_sum	Avg_medPressDuration	
22	Avg_nAutocorrect	AvgMedAB	AvgMedAB	
23	AvgMedBB	medianZ		
24	AvgVarBB	***nKeypresses***		
25	NoneOfTheseDiag	AvgMAD_AA		
26	autocorrectRate	Avg_nBackspace		
27	medianZ	count_Xhorizontal		
28	AvgVarAB	AvgVarAA		
29	autocorrectRate_wkSD	distToCenterPrevRatioAA		
30	medianX	Zmotion_sd		
31	medianIKD	Xmotion_sum		
32	Avg90PercentileAA	Avg90PercentileAA		
33	Ymotion_sum	medianY		
34	distToCenterPrevRatioAA	Ymotion_sd		
35	AvgMedAA			
36	Avg_nAlphanum			
37	AvgMedAB			
38	Ymotion_sd			
39	Xmotion_sd			
40	medIKD_wkSD			
41	Zmotion_sum			
42	Avg_medPressDuration			
43	medianY			
44	AvgVarAA			
45	bkspRate_wkSD			
46	SeasonalAffectiveDisorder			
47	Zmotion_sd			
48	Xmotion_sum			
49	Schizophrenia			
50	Diag_PreferNotAnswer			

^a^Features in bold/italic are used later in sensor fusion.

When all features were used for prediction, the clinical features dominated many of the top-ranking spots, with a few typing and accelerometer features such as medianPressDur and n_XYZ also showing significant contributions. Further comparing typing+accelerometer data to the slices for either typing only or accelerometer only, we note that medianPressDur, medianX, arc_sum, and n_XYZ consistently ranked in the top 5. These shifts across different slices of the data also influenced our choices for which features to attempt to use for sensor fusion, which we describe in the next session. Overall, these results indicate that there seems to be significant duplicative information in the different feature categories, which combined with the results in the Main Results section, again underscores the potential for using accelerometer data as a proxy for clinical data in individuals with bipolar disorder.

### Sensor fusion

Along with comparing different feature categories, we were also curious about what affect fusing features across categories might have, in particular, whether the model performance could be improved through such sensor fusion. To test this idea, we fused two accelerometer features—*medianX* (ranking #2), *arc_sum* (#4)—with four typing features—*medianPressDur* (#1), *backspaceRate* (#13), *autocorrectRate* (#15), *nKeypresses* (#24)—based on the results of feature selection using only typing and accelerometer data (the “Typing + Accel” column in Table 4). The accelerometer features were selected from those among the top ranks. For the typing features, one was chosen from the top rank (medianPressDur), whereas the other three were chosen based on their high degree of shifting between data slices (columns in Table 4), as well as their identified importance for predicting bipolar fluctuations in the previous research^6^.

Sensor fusion here was conducted by dividing each of the typing features by each of the accelerometer features to produce a single value (a ratio, in essence) that combined the information of both of the original features. After that, the original features were removed from the dataset, and only the new composite fused feature was used for modeling. Such modeling included performing the same feature selection and modeling process again as described in the Main Results and Feature Selection sections above, using the imputed dataset to evaluate the “Typing + Accel” features with the new fused feature (without clinical features). A total of eight different analyses were produced in this way so as to isolate the effects of each fusion. However, only four of those eight showed a higher feature ranking for the fused feature than the ranking for the original features (based on the average ranking of the two original features). For brevity, we only report the results of those four below, to see whether the improved fused feature led to any improvement in model performance or not.

### Fusion of “medianX” with typing features

Table 5 shows the results of sensor fusion using the medianX accelerometer feature with typing features that produced higher average feature rankings than the original features. Compared to the results of “Typing + Accel” in Table 2, the accuracy, AUC, and sensitivity are almost unchanged. We can see here that even if two features with high mutual information are fused as one, that still does not necessarily lead to higher predictive power in terms of model performance. Though we do note that the performance of the models in Table 2 was already quite good, so perhaps there was little room for improvement in this case.

**Table 5:** Sensor fusion of medianX feature with typing features.

Accelerometer	Classifier	Acc	Acc std	AUC	AUC std	Sensitivity
nKeypresses	Random Forest	0.9077	0.02	0.9386	0.001	0.9108
	Gradient Boost	0.8706	0.02	0.9347	0.01	0.8825
	Neural Network	0.8868	0.02	0.9426	0.01	0.9125
backspaceRate	Random Forest	0.9119	0.01	0.9449	0.001	0.9182
	Gradient Boost	0.8788	0.02	0.9396	0.02	0.9035
	Neural Network	0.8850	0.02	0.9435	0.02	0.9072

### Fusion of “arc_sum” with typing features

Similar to the previous section, Table 6 shows the results of sensor fusion using the arc_sum accelerometer feature with typing features that produced higher average feature rankings than the original features. Compared to the results of “Typing + Accel” in Table 2, the accuracy, AUC, and sensitivity are almost unchanged. The fusion did increase the rankings of the typing features (see next section), but once again, the overall performance was not enhanced in this case. Of course, it is possible other types of fusion on typing and accelerometer data from smartphones would see more success.

**Table 6:** Sensor Fusion of arc_sum feature with typing features.

Accelerometer	Classifier	Acc	Acc std	AUC	AUC std	Sensitivity
nKeypresses	Random Forest	0.9009	0.01	0.9406	0.01	0.9098
	Gradient Boost	0.8751	0.01	0.9417	0.02	0.8961
	Neural Network	0.8964	0.01	0.9494	0.01	0.9156
autocorrectRate	Random Forest	0.9017	0.02	0.9438	0.02	0.9129
	Gradient Boost	0.8767	0.03	0.9435	0.02	0.8909
	Neural Network	0.8958	0.02	0.9523	0.01	0.9131

### Feature selection of fused features

Table 7 shows the results of feature selection performed after sensor fusion for each of the four fusion attempts described earlier in this section. The most notable effect here was when combining nKeypresses and medianX. That shifted the combined feature to 3rd overall, whereas nKeypresses was originally 24th on the list. The combined feature was similar to the original medianX ranking (#2). This can be interpreted in a couple of ways: either the sensor fusion produced a more parsimonious dataset that could predict as well with fewer number of features or that nKeyPresses is not more relevant for predicting fluctuations in bipolar symptoms than medianX accelerometer alone regardless of whether it is fused or not.

**Table 7:** Top features after sensor fusion^a^.

Ranking	nKeypresses (24)/medianX (2)	Ranking	backspaceRate (13)/medianX (2)
1	medianPressDur	1	medianPressDur
2	count_Xhorizontal	2	n_XYZ
3	**nKeypresses_X**	3	count_Xhorizontal
4	autocorrectRate_wkSD	4	arc_sum
5	Avg_nAutocorrect	5	medianIKD
6	n_XYZ	6	**backspaceRate_X**
7	AvgVarBB	7	Zmotion_sum
8	backspaceRate	8	nKeypresses
9	AvgMedAB	9	medianDistCenter
10	AvgVarAB	10	AvgVarAA
11	medianZ	11	Avg_nAutocorrect
12	medianIKD	12	Avg_medPressDuration
13	Avg90PercentileAA	13	AvgVarAB

Ranking	nKeypresses (24)/arc_sum (4)	Ranking	autocorrectRate (15)/arc_sum (4)
1	medianPressDur	1	medianPressDur
2	count_Xhorizontal	2	n_XYZ
3	n_XYZ	3	count_Xhorizontal
4	AvgVarAB	4	medianX
5	medianDistCenter	5	medianDistCenter
6	Avg_nAutocorrect	6	**autocorrectRate_arc**
7	backspaceRate	7	bkspRate_wkSD
8	autocorrectRate	8	autocorrectRate_wkSD
9	medianIKD	9	Avg_medPressDuration
10	medianX	10	Avg_nBackspace
11	**nKeypresses_arc**	11	medIKD_wkSD
12	AvgVarAA	12	nKeypresses
13	AvgVarBB	13	medianIKD

^a^Features in bold are the newly fused features. For the original features at the top of each column, the number in parentheses is the original ranking from the “Typing + Accel” column in Table 4.

The other three sensor fusion analyses showed a similar pattern, compensating for the limitations of the typing features by fusing them with the accelerometer features. However, none of the fused features were better than the original accelerometer feature, nor did they improve overall model predictive performance (as noted in the previous sections above). Our overall takeaway from this analysis was that sensor fusion of the chosen features here had limited effect, and that using said features independently may be a better choice for the time being. Of course, it is possible that if the features are fused in a different way or if different typing and accelerometer features are chosen, then the result could be different. That is a question for future research, as there are essentially unlimited possibilities with regard to sensor fusion.

## Discussion

We analyzed a longitudinal open-science dataset of 291 individuals from the BiAffect smartphone app targeted at those with bipolar disorder, which contained rich details about their smartphone interactions (including typing patterns and accelerometer motion) collected everyday over several months, along with more traditional clinical features. The aim was to predict weekly fluctuations in depression symptoms in those individuals *prior* to the change. The main results showed that such smartphone data was capable of serving as a proxy for more traditional clinical data. Moreover, accelerometer data improved the predictive performance of machine learning models by nearly 5% over those previously reported in the literature based only on clinical data and typing dynamics. Our interpretation of these results is that some of the accelerometer features, notably median_X and arc_sum, may be related to characteristic patterns of psychomotor abnormalities such as agitation or impairment in bipolar disorder (see Introduction section). For instance, median_X may capture the frequency of side-to-side planar motion of the phone during use (i.e., swinging or swaying), while arc_sum might be indicative of the frequency of orientation changes of the phone (in a 3-dimension rotational sense).

A key takeaway from this study is that it appears that there are a number of parallel feature sets using different data related to bipolar disorder that can result in comparable predictive performance in machine-learning contexts. This harkens back to early days of researchers attempting to use machine learning to predict breast cancer genes in the early 2000s, including the famous arguments over the “optimal 70-gene set”^36–38^. Researchers gradually realized that there were actually many different sets of 70 genes that could obtain similar performance, given the complex interactions between genes and the small effects of any individual gene^39^. While that result was at first met with chagrin as a failure (given that all researchers want to be the one to “find the answer”), it eventually became accepted within the data science community that being able to solve a problem in multiple ways opens up opportunities to develop alternative solutions to a given problem as well as re-evaluate old solutions given new data, expanding our toolset. Not a failure per se, but rather an opportunity^40,41^.

Likewise, in the domain of mental health disorders, having multiple ways to model and predict patient trajectories expands our capabilities to deal with those problems in different ways in different settings^42^. For some patients, that might involve traditional clinical care, but for others, community-based rehabilitation may be more appropriate^43^. As the results show here, it is possible to elicit essentially the same “information” about the patients relative to their symptomology from different data sources, enabling us to track and monitor such patients in a variety of settings.

There were a number of limitations to this study, which are important to keep in mind. First, one major limitation was that the BiAffect app was designed to collect naturalistic data, via a crowd-sourced “open-science” approach (aka citizen science^44^). On the one hand, that means that our dataset here is more representative of the population that clinicians see during real-world practice, and that the “messiness” of the data is reflective of what one might find in a real-world clinical dataset. On the other hand, since that approach relies on self-reported data and patient-reported outcome measures (e.g., PHQ and MDQ), it lacks the rigorous validity of data that might be gathered in a controlled trial, though conversely, a controlled trial often results in less-representative data due to stringent inclusion criteria, strict protocols, and financial incentives for participants (none of which typically exist in real-world practice). That trade-off between research and practice is a long-standing one in healthcare that has been extensively discussed by LW Green and others^45^. Suffice it to say, there is likely a need for both types of research (naturalistic and controlled) in the field, but it’s important to note that both approaches have their limitations, which impact the generalizability of their results. That does suggest an opportunity for future research on the topic presented in this paper, to provide further lines of evidence.

Second, another limitation is that the BiAffect mobile app currently only works on iPhone, rather than Android as well. Part of that is a technical limitation in deploying these kinds of sensor-based *ambulatory assessment* apps on both iPhone and Android, due to the way their application programming interfaces (APIs) handle sensor hardware. Additionally, there are challenges with fragmentation in Android, with multiple concurrent OS versions and countless original equipment manufacturer (OEM) hardware modifications. Indeed, many existing crowd-sourced smartphone sensing studies tend to be done on iPhones only, for those very reasons^46^. There is an advantage to that approach, given that we do not have to deal with calibration issues across two platforms running different software. However, Android is available on a wider array of smartphone devices, including lower-cost devices. There are also vast differences in market share across countries. While wealthier countries like the United States, Canada, and Japan have roughly 50% iPhone market share, in many less-developed countries in Africa, South America, and Asia, conversely Android dominates the marketplace with 80–90% market share^47^. That means there may be some sample bias in our dataset that unintentionally excludes lower socioeconomic individuals. If, and how, any differences in typing dynamics or accelerometer data exist in such individuals is something that future research should consider. Doing so will likely demand *targeted* research aimed at lower-socioeconomic individuals and/or less-developed countries specifically, using Android-based data collection apps that are accessible to such populations, to overcome the limitations of the current generation of iPhone-based crowd-sourcing approaches.

Third, the use of the MDQ instrument here merits further discussion. While MDQ has been shown to be a valid screening questionnaire for bipolarity among patients with mood disorders with good overall diagnostic accuracy^48^, its use in the general population is less clear. In a recent large-scale study in England, MDQ was reported to have a lower sensitivity when it was applied to determine the lifetime prevalence of bipolarity in the community^49^. Indeed, due to the substantially lower prevalence of bipolar disorders in the general population (compared to, say, the prevalence among patients who present in psychiatric outpatient clinics), it becomes much harder for any screening test to have sufficient positive predictive value. However, it should be noted that in this study, many of the BiAffect app users who participated likely did so because of their personal connections to mental health and psychiatric disorders, and thus the study sample was drawn from a population enriched for mood disorders. For readers who are interested in the use of MDQ as a screening tool in different populations, we refer to recent discussions on its validity in refs. ^50,51^.

Finally, a fourth limitation here to consider is the cost and privacy risks associated with collecting this kind of data about individuals. Given that the BiAffect app used in this study is free for users largely mitigates the cost issue, but the privacy issues are ones that cannot be ignored. There is significant potential for typing dynamics and phone motion data (i.e., accelerometer) to be misused, or alternatively, if such data can be linked to individual behavior patterns or health symptoms, for that information to be used in ways that may not be in the best interest of the patient (e.g., creating individualized insurance premium rates). There is likely a need for some ethical standards and/or legal frameworks to be developed to regulate the use of this kind of smartphone data, similar to how the use of genetic information has been regulated in recent years^52,53^.

## Methods

### Dataset description

The target here was clinically-relevant changes in the PHQ outcome scale, defined as a difference of 4 or more points based on weekly sampling^35^. The aim was to predict those changes *before* they occur based on smartphone interaction data in the one week prior gathered via the BiAffect mobile app^4^. The dataset included three kinds of features: clinical, typing dynamics, and accelerometer (see Table 1). Our purpose here is to evaluate the utility of combining both typing and accelerometer data for making such predictions of the target, averse to previous studies that looked at only typing dynamics features^5,6^. Furthermore, we were interested in whether that combination would make it possible to exclude clinical data while still maintaining the high accuracy of the predictions. Excluding such clinical data might enable models that could be used to screen for bipolar-related symptoms in the general population, even amongst people who had never been diagnosed or those who had been diagnosed but not visited a doctor or clinic recently (and thus lack valid clinical data). The study was approved by the IRB at the University of Illinois—Chicago (protocol #2016-1261).

Before starting the modeling process, the data was pre-processed to deal with various data-related issues. All isomorphic features in the typing data, such as features that represent the forward and reverse order of the same feature (e.g., typing interval between alphabetical and backspace keys), were reduced to eliminate collinearity in the dataset so that only the forward-sequence intervals remained. After this, we were left with 50 features in total. Those features can be seen in Table 1, grouped by category. We note that for the accelerometer features, many are based on three-dimensional motion (defined as X, Y, and Z coordinates by the manufacturer), that can be visualized as shown in Fig. 1. More information can be found on IOS or Android websites, e.g., https://developer.apple.com/documentation/coremotion/getting_raw_accelerometer_events.

There were also many missing values in each column of data. The ratio of missing data generally ranged between 0% to up to 25–30% for some features, depending on the feature. As such, we attempted two ways to preprocess these data, with one approach replacing all the missing values with the average value across all individuals of the corresponding feature, and the other approach simply excluding all individuals with any missing data during analysis. For the latter case, excluding individuals resulted in a smaller dataset of 148 individuals, i.e., about half the individuals were missing some data. The results of both approaches are shown in the Results section.

On average, each individual had 9.6 weeks worth of data for analysis. A clinically-relevant PHQ change occurred roughly 18.7% of the time, with approximately half of the individuals experiencing at least one significant PHQ change (54.4%). The average age was 41.3, with nearly 66% being female. Roughly 2/3 of the individuals reported having been diagnosed bipolar and a similar number screened positive on the MDQ, whereas others were undiagnosed (who may or may not have bipolar). Approximately 62% reported having depression (with an average PHQ score of ~9.3), and 24% reported having PTSD. In terms of keypress dynamics, the average number of weekly keypresses per person was roughly 5280 (total of 15.1 million overall), while the average weekly autocorrect rate was 0.013 (1.3% of presses) and the backspace rate was 0.089 (8.9% of presses). The average interkey delay between keypresses was 360 milliseconds, whereas the typical hold time of each keypress (median press duration) was approximately 90 milliseconds.

### Analysis approach

Our primary analysis here looked at the comparison of different *slices* of the total dataset’s features described in the previous section. Those four slices can be categorized as shown below. We also note that a previously reported analysis of “Typing + Clinical” features using this same dataset showed similar performance as “Typing Features Only”^6^, so Typing + Clinical is omitted here for brevity.

1. All features (including clinical ones)
2. Typing and accelerometer features (excluding clinical features)
3. Typing features only
4. Accelerometer features only

For analyzing the data, multiple modeling methods were attempted: Random Forest, Gradient Boosting, and deep learning (DL) Neural Networks. Data here was primarily modeled using Python’s Scikit-Learn package (https://pypi.org/project/scikit-learn/). Models were run using the default parameters in Scikit, though some experimentation was performed (similar to ref. ^5^). For the neural networks, those were ran using the python package Keras (https://keras.io/, version 2.5), which is a deep learning library based on TensorFlow, using a single dense hidden layer with 70 units and sigmoid activation output layer. In previous research, we explored the effect of hyperparameter tuning the models (including varying the number and types of layers in the DL models), but in general, that had minimal effect on performance^6^. As such, models here are using set parameters based on that previous research. Beyond the models themselves, we also explored various types of feature selection to determine which features were driving patterns observed in the data. Those results are presented in the Results section. Model performance was estimated using multiple evaluation metrics, including accuracy (Acc) and area under the curve (AUC) based on fivefold cross-validation, following standard machine learning guidelines^54^.

Given the imbalanced nature of the target variable (~18.7% of weekly samples had a clinically-relevant change in PHQ score as defined at the beginning of the Methods section, versus 81% did not), we used Python’s imblearn package (https://pypi.org/project/imblearn/) to deploy a hybrid approach (combining undersampling with SMOTE^55^) in order to rebalance the data, based on its superior performance in the previous research^6^. Additionally, we evaluated multiple feature selection methods for comparison, including both filter-based and wrapper-based methods^56^. The filter-based method utilized information gain (i.e., entropy) to rank each feature (univariate approach) which could then be used to select some top k features (k-count). The wrapper-based method used a Random Forest model to evaluate different sets of features across hundreds of trials, identifying the best set of features based on the predictive performance of the resulting model. Furthermore, sensor fusion^13^ was utilized to evaluate whether directly combining typing dynamics and accelerometer features together into single features could lead to improved performance over the original raw features.

### Reporting summary

Further information on research design is available in the Nature Research Reporting Summary linked to this article.

## Supplementary information

Reporting Summary

## Data Availability

Data in de-identified form may be made available from the corresponding author upon reasonable request.

## Code availability

Code used here may be made available from the corresponding author upon reasonable request.
