# Supplementary material for: Smartphone accelerometer data as a proxy for clinical data in modeling of bipolar disorder symptom trajectory
Source: NPJ Digit Med. 2022 Dec 14;5:181. doi: 10.1038/s41746-022-00741-3 (PMC9751066; doi:10.1038/s41746-022-00741-3)
Supplement: Supplementary file 1 — Reporting Summary [file 41746_2022_741_MOESM1_ESM.pdf]

nature portfolio

Corresponding author(s): Casey C. Bennett
Manuscipt ID: NPJDIGITALMED-04290R

Last updated by author(s): Dec 2, 2022

Reporting Summary

Nature Portfolio wishes to improve the reproducibility of the work that we publish. This form provides structure for consistency and transparency in reporting. For further information on Nature Portfolio policies, see our Editorial Policies and the Editorial Policy Checklist.

Statistics

For all statistical analyses, confirm that the following items are present in the figure legend, table legend, main text, or Methods section.

n/a	Confirmed	
☐	☒	The exact sample size (*n*) for each experimental group/condition, given as a discrete number and unit of measurement
☐	☒	A statement on whether measurements were taken from distinct samples or whether the same sample was measured repeatedly
☐	☒	The statistical test(s) used AND whether they are one- or two-sided<br>*Only common tests should be described solely by name; describe more complex techniques in the Methods section.*
☒	☐	A description of all covariates tested
☒	☐	A description of any assumptions or corrections, such as tests of normality and adjustment for multiple comparisons
☒	☐	A full description of the statistical parameters including central tendency (e.g. means) or other basic estimates (e.g. regression coefficient) AND variation (e.g. standard deviation) or associated estimates of uncertainty (e.g. confidence intervals)
☐	☒	For null hypothesis testing, the test statistic (e.g. *F*, *t*, *r*) with confidence intervals, effect sizes, degrees of freedom and *P* value noted<br>*Give P values as exact values whenever suitable.*
☒	☐	For Bayesian analysis, information on the choice of priors and Markov chain Monte Carlo settings
☒	☐	For hierarchical and complex designs, identification of the appropriate level for tests and full reporting of outcomes
☒	☐	Estimates of effect sizes (e.g. Cohen's *d*, Pearson's *r*), indicating how they were calculated

Our web collection on statistics for biologists contains articles on many of the points above.

Software and code

Policy information about availability of computer code

Data collection	Data was collected via the BiAffect mobile app, which is described in the paper
Data analysis	Data analysis was conducted in Python, using open-source machine learning and deep learning libraries (Scikit-Learn, Keras, Tensorflow)

For manuscripts utilizing custom algorithms or software that are central to the research but not yet described in published literature, software must be made available to editors and reviewers. We strongly encourage code deposition in a community repository (e.g. GitHub). See the Nature Portfolio guidelines for submitting code & software for further information.

Data

Policy information about availability of data

All manuscripts must include a data availability statement. This statement should provide the following information, where applicable:

- Accession codes, unique identifiers, or web links for publicly available datasets
- A description of any restrictions on data availability
- For clinical datasets or third party data, please ensure that the statement adheres to our policy

Data in de-identified form may be made available from the corresponding author upon reasonable request.

Human research participants

Policy information about studies involving human research participants and Sex and Gender in Research.

Reporting on sex and gender	Details on the sample, including Gender, are provided in the Methods section
Population characteristics	Details on the sample, including Age are provided in the Methods section
Recruitment	Participants could sign up via a mobile app available in the app store, promoted for use in monitoring mood and cognition, such as in Bipolar Disorder. The data was "crowd-sourced" using an open-science approach, to collect naturalistic data.
Ethics oversight	University of Illinois - Chicago (Protocol #2016-1261)

Note that full information on the approval of the study protocol must also be provided in the manuscript.

Field-specific reporting

Please select the one below that is the best fit for your research. If you are not sure, read the appropriate sections before making your selection.

☒ Life sciences ☐ Behavioural & social sciences ☐ Ecological, evolutionary & environmental sciences

For a reference copy of the document with all sections, see nature.com/documents/nr-reporting-summary-flat.pdf

Life sciences study design

All studies must disclose on these points even when the disclosure is negative.

Sample size	n=291 ... we use a crowd-sourced "open-science" approach for sampling via the BiAffect mobile app, in order to collect a naturalistic dataset for machine learning purposes. There was no predetermined sample size, or statistical comparisons between groups.
Data exclusions	There were no exclusions, other than excluding participants who only used the mobile app for less than 1 month (i.e. due to insufficient data).
Replication	We evaluated the dataset using multiple machine learning and deep learning methods to see whether the results were consistent (which they were). Additionally, there are previous publications with both the BiAfffect sample as well as from other researchers using different samples from other apps, which have produced similar results. Several of those papers are all cited in the the first paragraph of the paper.
Randomization	Not relevant. There were no experimental groups.
Blinding	Not relevant. There were no experimental groups.

Reporting for specific materials, systems and methods

We require information from authors about some types of materials, experimental systems and methods used in many studies. Here, indicate whether each material, system or method listed is relevant to your study. If you are not sure if a list item applies to your research, read the appropriate section before selecting a response.

Materials & experimental systems

n/a	Involved in the study	
☒	☐	Antibodies
☒	☐	Eukaryotic cell lines
☒	☐	Palaeontology and archaeology
☒	☐	Animals and other organisms
☒	☐	Clinical data
☒	☐	Dual use research of concern

Methods

n/a	Involved in the study	
☒	☐	ChIP-seq
☒	☐	Flow cytometry
☒	☐	MRI-based neuroimaging
